# Perception and Attitude Towards COVID-19 Vaccination Among the Elderly: A Community-Based Cross-Sectional Study

**DOI:** 10.7759/cureus.33108

**Published:** 2022-12-29

**Authors:** Chirag Sandooja, Jugal Kishore, Aninda Debnath, Aftab Ahmad

**Affiliations:** 1 Department of Community Medicine, Vardhman Mahavir Medical College and Safdarjung Hospital, Delhi, IND

**Keywords:** covaxin, covishield, elderly patients, sars-cov-2 (severe acute respiratory syndrome coronavirus -2), coronavirus disease, kap study, cross sectional studies, elderly population, covid-19 vaccine, covid 19

## Abstract

Introduction

COVID-19 is one of the most formidable obstacles that humanity has encountered in this century. The death rate was high among the elderly in India; therefore, getting the elderly vaccinated was one of the most important things to do.

Objective

We conducted this study to assess the perception and attitude about the COVID-19 vaccines among the elderly population.

Methods

This cross-sectional study was conducted at Fatehpur Beri, New Delhi. We selected 108 participants using systematic random sampling. We used a semi-structured questionnaire to collect the data.

Results

Out of 108 participants, 52.8% were men. Among them, 9.3% of participants had tested positive before. The average number of days of illness among the participants was 5.3 (SD + 3.5). Males had a higher average day of illness (5.5, SD +3.7) than females (4.9, SD +3.3). Among those who had not been vaccinated, 73.3% of participants said they would receive the vaccine, 6.7% were unsure, and 20% were not willing to receive the vaccine.

Conclusion

COVID vaccination in an elderly population showed a relatively high vaccine acceptance rate, and the willingness to get the vaccine was also high among the unvaccinated.

## Introduction

Currently, COVID-19 is one of the most challenging problems that people have had to deal with in this century. As of September 30, 2022, the disease had caused over six million deaths and 614.7 million incidents worldwide [1]. By September 30, 2022, India had recorded 44.5 million cases with 528,629 deaths [2]. Many initiatives have been made globally to combat the pandemic. The efforts made in India, a country with scarce resources, have likewise been multi-dimensional [3].

During the current COVID-19 epidemic, it was imperative to use a COVID-19 vaccine that is both safe and effective. Even though many epidemiologists and other medical professionals have concerns and questions about how quickly the COVID-19 vaccine was approved, many people are still hopeful that it will work. Research suggests that the vaccine created by Pfizer and BioNTech can protect over 90% of patients from the severity and mortality of COVID-19. However, the short-term effectiveness study for the Moderna vaccine calls for 94.5% protection [4,5].

The first wave of the COVID-19 virus that hit India projected a high mortality rate among people aged 60 and older, particularly those who already were suffering from several health conditions. Therefore, the vaccination of the elderly population was a top priority in India. The successful development and use of vaccines have given people hope that the COVID-19 pandemic can be stopped. However, this depends on how widely available vaccines are and how willing people are to accept and use them [6]. Although vaccines are generally created and distributed to mitigate the consequences of the pandemic, there is evidence that people are reluctant to accept vaccines globally [7]. The success of vaccination efforts that try to generate herd immunity through mass vaccination mainly depends on the public's attitude toward and perception of available vaccinations [8,9]. The perception and attitude of the elderly toward vaccines remain unexplored. This study assessed the perception and attitude about the COVID-19 vaccines among the elderly population.

## Materials and methods

Study type and setting

We conducted this cross-sectional survey among the elderly (aged more than 60 years) residing at Fatehpur Beri, Delhi, from August to October 2021.

Sample size

The sample size for the study was 108. As no previous study assessed the acceptance of the COVID-19 vaccine among the elderly, we calculated the sample size by taking a prevalence of 50%, a relative error of 20%, and a 10% non-response rate. The inclusion criteria included those older than 60 years of age and residing in Fatehpur Beri for more than six months. We used systematic random sampling to select the participants. The village was divided into four parts, and from each part, 27 households were selected. Every nth house was selected until the sample size was achieved. We visited the household and selected the study participant using the Kish selection table.

Study tool

We used a semi-structured questionnaire to collect the data. The field experts validated the questionnaire to increase its face validity. The semi-structured questionnaire had two parts. The first part had sociodemographic information about the participants and characteristics of COVID-19 disease, and the other parts included perceptions and attitudes about the COVID-19 vaccine. In the later part, there were three sections regarding knowledge, attitude, and practice of COVID-19 vaccination.

Statistical analysis

Data analysis was done using IBM's Statistical Package for Social Sciences (SPSS) for Windows (v21.0). Categorical data were presented as percentages, and Pearson's chi-square test was used to assess significant differences between groups. If the expected number in a cell was found to be less than five, Fisher's exact test was used. Normally distributed continuous data were presented as a mean and standard deviation. All tests were performed with an alpha error rate of 5%; thus, a value less than 0.05 (p-value 0.05) was considered statistically significant.

Ethics clearance

Ethics clearance was obtained from the Institute Ethics Committee, Vardhman Mahavir Medical College (VMMC) and Safdarjung Hospital (Ref. no. IEC/VMMC/SJH/Thesis/2020-11/CC-77). Written informed consent was obtained from all the participants.

## Results

Out of 108 participants, 52.8% were males. Most participants (81.5%) were between the ages of 60 and 65. Most study participants (63.9%) had never had a reverse transcription polymerase chain reaction (RT-PCR) or rapid antigen test (RAT) to test for COVID-19 (57.4%). Out of the 39 people who underwent RT-PCR testing, 10 (25.6%) participants tested positive. Rapid antigen testing was performed on 46 participants, and 25 (54.3%) had positive results (Table 1).

**Table 1:** COVID-19 testing status among the participants (n=108)

	Male, n (%)	Female, n (%)	Total N (%)
Total	57 (52.8%)	51 (47.2%)	108 (100%)
RT-PCR test			
RT-PCR positive	6 (10.5%)	4 (7.85)	10 (9.3%)
RT-PCR negative	15 (26.3%)	14 (27.5%)	29 (26.9%)
Never underwent RT-PCR testing	36 (63.2%)	33 (64.7%)	69 (63.9%)
Rapid antigen test (RAT)			
Rapid antigen test positive	19 (33.3%)	6 (11.7%)	25 (23.2%)
Rapid antigen test negative	7 (12.3%)	14 (27.5%)	21 (19.4%)
Never underwent RAT	31 (54.4%)	31 (60.8%)	62 (57.4%)
COVID status			
COVID positive (RT-PCR)	22 (38.6%)	8 (15.7%)	30 (27.8%)
COVID negative	35 (61.4%)	43 (84.3%)	78 (72.2%)

The participants' average number of days of illness was 5.3 (SD+ 3.5) days. We calculated the duration of the illness from the day of the diagnosis to the final day of symptoms. Males had a higher average day of illness (5.5, SD+3.7) than females (4.9, SD +3.3). The majority of study participants were symptomatic. Only 16.7% of study participants had no symptoms of COVID. Fever was the most common symptom reported by the participants. Fever affected 83.3% of the participants. Fever affected 86.4% of the male participants and 75% of the females. Other symptoms, such as diarrhea, loss of smell, and body aches, were experienced by only 10% of the participants. Only the male participants experienced these symptoms; none of the female participants did (Table 2).

**Table 2:** Characteristics of COVID-19 symptoms among participants

	Male n (%)	Female n (%)	Total N (%)	p-value
The average day of illness because of COVID-19 (mean, SD)	5.5 (3.7)	4.9 (3.3)	5.3 (3.5)	0.42
Did the patient have symptoms of COVID-19?				
Yes (n%)	19 (86.4%)	6 (75.0%)	25 (83.3%)	0.54
No (n %)	3 (13.6%)	2 (25%)	5 (16.7%)	
Did the patient have a fever?				
Yes (n %)	19 (86.4%)	6 (75.0%)	25 (83.3%)	0.46
No (n %)	3 (13.6%)	2 (25%)	5 (16.7%)	
Other symptoms (diarrhea, loss of smell, body ache)				
Yes (n %)	3 (13.6%)	0 (0%)	3 (10%)	0.27
No (n %)	19 (86.4%)	8 (100%)	27 (90%)	
Vaccination status				
Had COVID before vaccination (n %)	14 (63.6%)	6 (75%)	20 (66.7%)	0.55
Got COVID after vaccination (n %)	8 (36.4%)	2 (25%)	10 (33.3%)	

**Figure 1:**
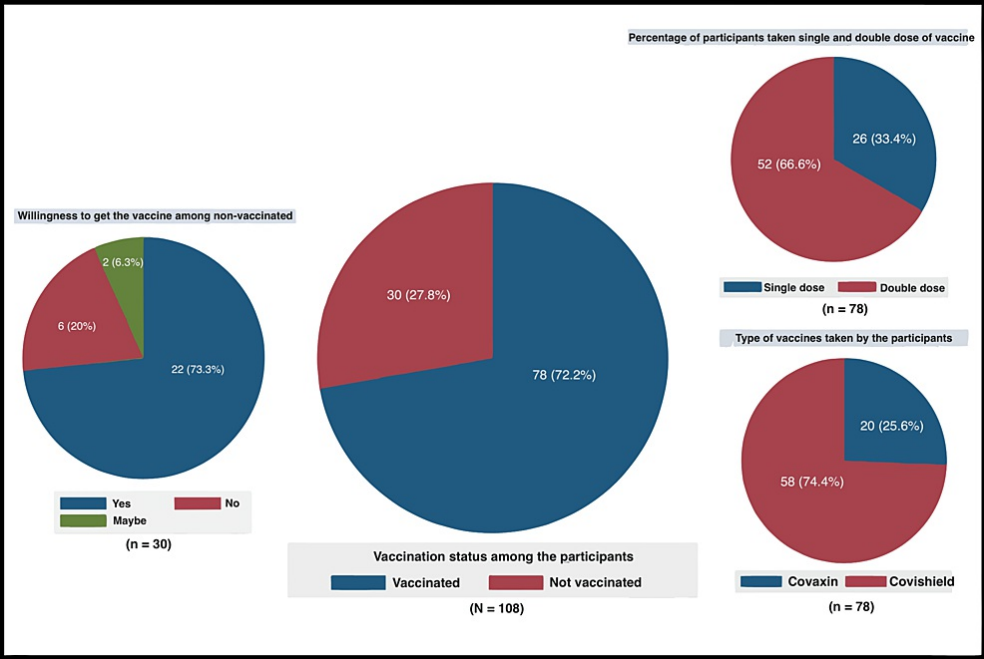
Vaccination status among the participants

Among the 108 study participants, 78 (72.2%) were vaccinated, whereas 30 (27.8%) were not. Among males, 43 (75.4%) were vaccinated, and 35 (68.6%) females were vaccinated. Ten (33.3%) of the 30 people who developed COVID-19 contracted it after vaccination, while the remaining 66.7% of subjects who got COVID-19 were not vaccinated. At that time, 66.6% of the vaccinated population had received a double vaccine dosage. Nearly three-quarters of individuals, 58 (74.4%), were vaccinated with Covishield, while the remaining 25.6% were vaccinated with Covaxin. Among those who had not been vaccinated, 73.3% of participants were willing to receive the vaccine, 6.3% were unsure, and 20% were not willing to receive the vaccine (Figure 1). Based on the study participants, 25% had no symptoms after vaccination, 26.9% had a fever after vaccination, and 27.8% had other symptoms after vaccination (Figure 1).

Seventy-five percent of participants believe that the COVID-19 vaccine can protect against infection, whereas 8% disagree, and others have not commented. Regarding the safety of the vaccination, 95 (87.9%) participants considered the vaccine safe for human use, while 6.5% of respondents had concerns about the vaccine's safety. Most participants (89.8%) wanted their child or spouse vaccinated. Seven people were hesitant to vaccinate their child or spouse. More than half of the participants (53.7%) reported that they would still undergo vaccination despite having recovered from COVID. One hundred and six participants (98.2%) said vaccines should be provided at no cost. Approximately 75% of the individuals used Unani or ayurvedic immunity boosters as a preventative measure. Moreover, only 3.7% of them used vitamin D3 and ivermectin as prevention methods (Table 3).

**Table 3:** Participants' attitudes toward the vaccine

Attitudes toward the vaccine among participants	Yes n (%)	No n (%)	Don’t know n (%)
Percentage of participants who thought people can be protected from COVID -19, by vaccination	81 (75%)	8 (7.4%)	19 (17.6%)
Percentage of participants who considered the COVID-19 vaccine to be safe	95 (87.9%)	7 (6.5%)	6 (5.6%)
Percentage of participants who were willing to get their child or spouse vaccinated	97 (89.8%)	7 (6.5%)	4 (3.7%)
Percentage of participants who were willing to go for vaccination if they have already got COVID-19 and recovered	58 (53.7%)	24 (22.2%)	26 (24.1%)

## Discussion

This study was conducted to learn about the knowledge, attitude, and practice of the COVID-19 vaccine among the elderly population of Fatehpur Beri, Delhi. It was observed that most participants were well aware of the COVID-19 vaccine and were likely to receive it if it were available. Among participants, men were 52.78% and women were 47.22%. The majority of participants were aged 60-65 (81.48%). RT-PCR and even rapid antigen tests were not done by most patients. Higher vaccination acceptance in this study demonstrates positive public confidence in the COVID-19 vaccine. There may be many reasons for this in the Indian subcontinent. One of the reasons is that vaccination provision and acceptance of vaccines in India are high due to the efforts of the Indian government's collaboration with UNICEF and the Global Alliance for Vaccines and Immunization (GAVI) in providing free vaccines and universal immunization. The vaccines were free, contributing to the high vaccine intake. The multimodal approaches to vaccine promotion also acted as a positive catalyst for high vaccine uptake.

The majority of the people, 83.3%, had symptoms of COVID. The most common symptom was fever. All the symptomatic patients had fevers. A study by Patgiri PR et al. found coughing to be the most common symptom, followed by fever and breathing difficulty [10]. Another study by Salins N et al. shows that 81% of the elderly had mild symptoms of COVID [11]. This result closely resembles the findings of our study. In our study, most of the symptoms were restricted to only fever. Severe symptoms, such as breathlessness, were less common in our study. These variances in symptoms may be attributed to the period of the study when it was conducted.

The average duration of illness due to COVID was 5.3 days (SD+3.5 days). The duration of the disease was longer in males compared to females. However, the difference was not statistically significant. In a study conducted in the United States by Tenforde MW et al., the average duration of symptoms is five to 10 days [12]. Similar to our study, this study also found that the symptoms of those who reported fever and chills on the day of testing resolved in 97% and 96% of respondents, respectively. The study conducted by Barman MP et al. showed that the estimated time of recovery was 25 days for the elderly [13]. Nevertheless, this study also states that the duration of illness among males is longer than among females. The study was conducted in the initial phase of COVID, so the isolation period was longer among those patients. Another reason for this variation might be the change in the COVID-19 variants. Different variants of COVID have different clinical symptoms, which also explains the various symptoms.

Among the 108 study participants, 72.2% were vaccinated. Among the 78 vaccinated people, 10 (12.8%) developed COVID-19. Whereas, among the 30 unvaccinated persons, 20 (66.7%) developed COVID-19. Of those who were vaccinated, 60% of the people were symptomatic. Moreover, among the unvaccinated, 95% were asymptomatic. A study conducted by Arora G et al. [14] shows that among the vaccinated, 7.3% of people developed COVID-19, and among the unvaccinated, 54.9% developed COVID-19. This finding was similar to our study. The difference that exists may be due to the fact that the author used a larger sample size. Contrary to our study, the percentage of symptomatic patients is higher in vaccinated patients compared to unvaccinated patients. Another study done in India by Kaur U. et al. shows similar patterns as ours. Among the vaccinated, 70% of the participants were symptomatic, and for those who were not vaccinated, 85.3% were symptomatic [15].

Nearly three-quarters of individuals, 58 (74.4%), were vaccinated with Covishield, while the remaining 25.6% were vaccinated with Covaxin. This replicated the national ratio, and this may be due to the easier availability of Covishield compared to Covaxin. Most of the unvaccinated (73.3%) were willing to take the vaccine. A study conducted among the elderly in China shows that 79.1% of the elderly were willing to take the vaccine [16]. The study in India by Jacob J. et al. shows that 78.6% of the elderly were willing to take the vaccine [17]. Our prevalence of vaccine acceptance was similar to a population-based study conducted in France, Denmark, Australia, Mexico, and Ireland [18-21]. Studies conducted in the UK (63.5%-67%), Saudi Arabia (64.7%), and Italy (53.7%) showed a lower prevalence of vaccine acceptance compared to our study [22-24]. In our study, we found that 20% of people were unwilling to vaccinate. Moreover, similar to this study, the reasons were fear of the vaccine's side effects, lack of trust in the existing healthcare system, and information gaps, which are very common in a low- and low-middle-income country (LMIC) setting.

Our study found that one-fourth of the patients had no symptoms after vaccination, and 26.9% had a fever after the COVID-19 vaccination. Similar findings were shown by studies that were conducted in India [25]. A study in Bangladesh has shown that 50.8% of the participants had some side effects following vaccination. We found that the most common side effect in this study was fever. 26.9% of the people developed a fever after vaccination. Other studies have reported pain and swelling at the injection site and fever as the most common adverse effects following vaccination [23,26].

It was found that 75% of the participants believed in the vaccine's efficacy. The studies conducted in Nepal (78.4%) and China (75.8%) reported similar or higher results [27,28]. Studies conducted in Pakistan and Ethiopia showed that people's perception of vaccine effectiveness was relatively low. Pakistan, where it was 47.7%, and Ethiopia, where it was 41.1%. In our study, 87.9% of participants thought the vaccine was safe for human use. In China (76%), Ethiopia (71%), and Nepal (64.5%), people believed that the vaccine was safe [29,30].

Limitations

The study was conducted using a questionnaire, but a qualitative approach would have been better suited to assess their knowledge, attitude, and perception about COVID-19 vaccination.

## Conclusions

This study assessed the general attitudes and perceptions of COVID vaccination in an elderly population, demonstrating a relatively high vaccine acceptance rate. To prepare for promoting vaccination with currently available vaccines and potential newly-developed vaccines against future emerging diseases, relevant governments and vaccination authorities should launch routine health education and campaigns to improve public perception and knowledge of vaccination in general and to ensure an optimal vaccination experience.
